# Assessing Adherence: Audit of Glasgow Antipsychotic Side-Effect Scale Completion for Patients on Antipsychotic Depot Injections at Guildford CMHRS SABP

**DOI:** 10.1192/bjo.2024.435

**Published:** 2024-08-01

**Authors:** Hardeep Singh, Maryam Syed, Vimal Mannali, Ekta Sharma

**Affiliations:** Surrey and Borders NHS Foundation Trust, Surrey, United Kingdom

## Abstract

**Aims:**

To evaluate the current use of the GASS scale in monitoring patients on antipsychotic depot medications at Guildford CMHRS, we conducted a comprehensive assessment and compared it with trust guidelines. The guidelines recommend GASS scale completion at specific intervals: 12 weeks and 6 months post-initiation, annually thereafter, and at each dose titration.

**Methods:**

We conducted a review of patient depot charts and SystmOne notes for individuals on antipsychotic depot medications, in accordance with trust guidelines. Cases were randomly selected from the total number of patients on these medications. Following the initial audit, which was shared at Guildford CMHRS, a recommendation was made for staff to utilize the GASS Scale during outpatient appointments and document scores on both charts and SystmOne. A follow-up audit after six months was performed to evaluate any improvements.

**Results:**

In the initial audit of 60 cases receiving antipsychotic depot injections, GASS was conducted in 8 cases (26.6%), with 7 cases (23.3%) completed within the last year. In the re-audit of 58 cases, GASS was completed in 16 cases (55.17%), all within the last year.

**Conclusion:**

The re-audit highlights a notable increase in completion rates, yet opportunities for improvement persist. Additional suggestions for enhancing completion encompass regular refresher courses on Trust Guidelines, ensuring Pharmacy Team adherence to guidelines for patients on antipsychotics, and motivating medics and nursing staff to complete and document the GASS Scale. Consistent re-auditing is recommended for continuous improvement.

